# Xanthine Oxidase Inhibitory Potential of *Achillea santolina* Methanolic Extract with ROS Modulation in HepG2 Cells

**DOI:** 10.3390/ijms27083401

**Published:** 2026-04-10

**Authors:** Arwa R. Althaher, Mirna W. Awadallah, Muhammad N. Qattoum

**Affiliations:** Department of Pharmacy, Faculty of Pharmacy, Al-Zaytoonah University of Jordan, Amman 11733, Jordan

**Keywords:** *Achillea santolina*, LC–MS, xanthine oxidase, reactive oxygen species, flavonoids, antioxidant activity

## Abstract

*Achillea santolina* is traditionally utilized in herbal therapy; nonetheless, its xanthine oxidase inhibitory and cellular antioxidant properties are not extensively investigated. This work aimed to identify the phytochemical composition of the methanolic extract of *A. santolina* aerial parts via LC–MS and to assess its xanthine oxidase (XO) inhibitory activity and its modulatory effects on intracellular reactive oxygen species (ROS) in HepG2 cells. The methanolic extract underwent LC–MS analysis for phytochemical identification, and XO inhibitory activity was assessed spectrophotometrically using allopurinol as the reference medication. Intracellular ROS levels were determined using the DCFH-DA fluorescent assay following xanthine-induced oxidative stress. LC–MS profiling identified ten compounds representing 95.1% of the extract, with flavonoid glycosides (44.1%) as the predominant class. Rutin (18.6%) and luteolin-7-O-glucoside (15.2%) were the major constituents. The extract demonstrated concentration-dependent inhibition of XO, with an IC_50_ value of 29.3 ± 0.4 µg/mL, compared to 2.1 ± 0.3 µg/mL for allopurinol. In HepG2 cells, xanthine significantly increased ROS production, while pre-treatment with the extract greatly diminished ROS levels in a dose-dependent manner, reaching approximately 75% inhibition at 50 µg/mL. The results demonstrate that the methanolic extract of *A. santolina* exhibits significant xanthine oxidase inhibitory and antioxidant properties, possibly due to its elevated flavonoid concentration, indicating its potential utility in addressing oxidative stress-related disorders. To the best of our knowledge, this study provides the first report on the combined xanthine oxidase inhibitory and intracellular ROS-modulating activities of *A. santolina* methanolic extract.

## 1. Introduction

The excessive generation of reactive oxygen species (ROS) induces oxidative stress, which plays a role in the development of several metabolic and inflammatory disorders [1]. Xanthine oxidase (XO) is a crucial enzyme in purine metabolism, catalyzing the transformation of hypoxanthine to xanthine and xanthine to uric acid [2]. The overactivation of xanthine oxidase induces hyperuricemia and elevates oxidative stress, resulting in tissue damage and the progression of disease [3].

Natural products are rich in bioactive compounds that function as antioxidants and enzyme inhibitors. Phenolic acids and flavonoids have been found to be effective free radical scavengers as well as inhibitors of xanthine oxidase (XO) [4]. Medicinal plants rich in these phytochemicals are thus shown as promising candidates for the development of safer medicinal drugs. Several plant-derived phytochemicals have been reported to be effective XO inhibitors, including green tea polyphenols [5], blueberry flavonoids [6], and grape stilbenes [7]. These naturally occurring compounds may provide therapeutic benefits while causing fewer negative effects than common synthetic inhibitors like allopurinol.

Flavonoids are one of the most important types of plant-derived polyphenols, with various biomedical applications. These molecules have a variety of pharmacological properties, including antioxidants [8], anti-inflammatory [9], anticancer [10], and cardioprotective effects [11]. Their biological effects are mainly due to their ability to scavenge reactive oxygen species, bind metal ions, and alter key cellular signaling pathways associated with oxidative stress and inflammation [12]. Furthermore, flavonoids have been shown to interact with numerous enzymes, including xanthine oxidase, lowering uric acid generation and oxidative damage [13]. Flavonoids are attractive options for the development of safer therapeutic agents targeting oxidative stress-related disease, due to their low toxicity and multifunctional biological activity [12].

Recent research has provided mechanistic insight into flavonoids’ structure-activity relationships, with an emphasis on differences between glycosylated and aglycone forms [14].

Flavonoid glycosides are generally more soluble and accessible, although aglycones have a stronger direct enzyme inhibitory effect due to better interaction with active sites [15]. Moreover, both subclasses have been demonstrated to modulate intracellular oxidative stress by various methods, including direct ROS scavenging, antioxidant enzyme modulation, and suppression of ROS-producing systems such xanthine oxidase [16]. A recent study found that structural features such as hydroxylation patterns and glycosylation significantly influence flavonoid-mediated enzyme inhibition and cellular redox modulation [17]. These findings highlight the importance of phytochemical composition in regulating biological activity and support the investigation of flavonoid-rich extracts, which have a dual role in enzyme inhibition and oxidative stress regulation.

*Achillea santolina* L. (Asteraceae) is a perennial aromatic herb used in folk medicine in the Mediterranean and Middle East [18]. The genus *Achillea* contains roughly 140 species that are extensively spread throughout Europe, Asia, and North Africa [19], with many of them known for their therapeutic properties. *Achillea* species have traditionally been used as natural treatments for wound healing, hemorrhage, cephalalgia, inflammation, spasms, flatulence, and gastrointestinal ailments [20]. 

*Achillea* species are rich in bioactive constituents, particularly terpenoids, flavonoids, and phenolic acids [21,22], which are associated with diverse pharmacological activities such as hepatoprotective [23], antioxidant [24], antimicrobial [25], and antifungal [26], anti-inflammatory [27], analgesic [28], and antispasmodic effects [29]. Despite the well-documented medicinal potential of *Achillea* species, limited information is available regarding the specific phytochemical composition of *A. santolina* and the combined evaluation of its xanthine oxidase inhibitory activity and cellular antioxidant potential. Therefore, the present study designed to characterize the phytochemical constituents of the methanolic extract of *A. santolina* aerial parts using LC–MS and to investigate its xanthine oxidase inhibitory activity as well as its ability to modulate intracellular reactive oxygen species levels in HepG2 cells.

This work is, to our knowledge, the first thorough inquiry that integrates LC–MS-based phytochemical profiling with xanthine oxidase inhibitory assessment and intracellular ROS modulation examination of *A. santolina* methanolic extract. This current method offers new perspectives into the dual functional role of this plant in enzyme inhibition and the regulation of cellular oxidative stress, hence underscoring its promise as a natural therapeutic candidate for illnesses connected to oxidative stress.

## 2. Results

### 2.1. Phytochemical Analysis

**Figure 1:**
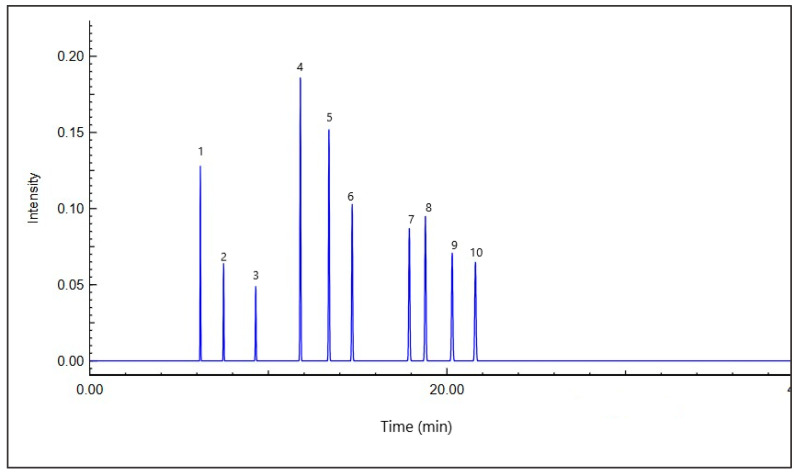
The chromatogram of *A. santolina* methanolic extract. Peaks are numbered according to their elution order and correspond to compounds identified by GC-MS analysis.

The chemical composition of the methanolic extract from the aerial parts of *A. santolina* was analyzed using LC–MS. Table 1 summarizes the identified phytochemicals along with their retention times, peak area percentages, and chemical classifications, while Figure 1 shows the corresponding LC–MS chromatograms.

Phytochemical profiling of the extract revealed the presence of ten compounds with one unidentified peak, representing a total identification of 95.1% of the extract composition. The detected components were primarily phenolic compounds, which included non-flavonoid phenolic acids and flavonoids. Chlorogenic acid and caffeic acid were found to be non-flavonoid phenolic acids, while the remaining components are flavonoids and glycosides.

Among the identified compounds, rutin (18.6%) was the main constituent, followed by luteolin-7-O-glucoside (15.2%). While kaempferol (6.5%), and caffeic acid (6.4%) were present in low amounts.

According to chemical classification, flavonoid glycosides made up the highest percentage (44.1%), followed by flavonoid aglycones (31.8%), showing that flavonoids are the predominant phytochemical class in the methanolic extract.

On the other hand, compounds identification is based mainly on LC-MS spectrum matching and literature comparison. The related structures remain theoretical in the absence of detailed MS/MS fragmentation data and valid reference standards.

### 2.2. Xanthine Oxidase Inhibitory Activity

The methanolic extract of *A. santolina* was tested for xanthine oxidase inhibitory activity and compared to allopurinol, a standard medication. Methanolic extract and allopurinol demonstrated concentration-dependent inhibition, with activity gradually increasing from 5 to 100 µg/mL.

**Figure 2:**
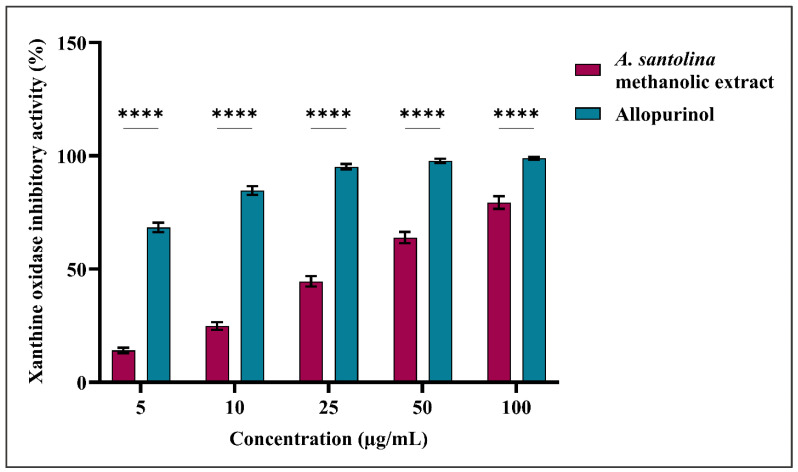
Xanthine oxidase inhibitory activity (%) of *A. santolina* methanolic extract and Allopurinol (standard reference) across concentrations of 5–100 µg/mL. Data are expressed as mean ± SD (*n* = 3), with error bars representing standard deviation. Statistical significance relative to the control is indicated as **** *p* < 0.0001.

At a concentration of 5 µg/mL, the methanolic extract inhibited xanthine oxidase by 14.1 ± 1.2% and allopurinol by 68.4 ± 2.1%. At 100 µg/mL, the extract enhanced activity to 79.4 ± 2.8%, while allopurinol inhibited activity by 98.9 ± 0.6% (Figure 2).

The IC_50_ result for the methanolic extract was 29.3 ± 0.4 µg/mL, while allopurinol revealed a significantly lower IC_50_ of 2.1 ± 0.3 µg/mL, indicating the greater potency of the standard (**** *p* < 0.0001 compared to the extract).

### 2.3. Effect of A. santolina Extract on ROS Levels

Treatment with xanthine alone significantly increased intracellular ROS production in HepG2 cells compared with the untreated control group (*p* < 0.001), confirming the induction of oxidative stress through xanthine oxidase activity. Intracellular ROS levels were calculated using the fluorescence intensity obtained from the DCFH-DA test. ROS levels were normalized to the untreated control group at 100%, and all treated groups were displayed as percentages % of this baseline. Pre-treatment with *A. santolina* methanolic extract reduced ROS formation significantly in a concentration-dependent manner. ROS levels dropped by 18 ± 1.5% at 5 µg/mL compared to the xanthine-treated group. Treatment with 10 µg/mL and 25 µg/mL resulted in decreases of 35 ± 2.0% and 62 ± 2.8%, respectively. Increasing the extract concentration to 50 µg/mL resulted in a stronger inhibitory effect, lowering ROS levels by 75 ± 3.1%. The observed decreases were statistically significant at all tested doses (Table 2).

These results show that the methanolic extract significantly decreases intracellular ROS accumulation in HepG2 cells, indicating significant antioxidant potential due to its xanthine oxidase inhibitory activity.

## 3. Discussion

Oxidative stress contributes significantly to the development and progression of hyperuricemia by increasing inflammatory responses and disrupting uric acid metabolism [30]. Many medicinal plants contain bioactive phytochemicals such as flavonoids, phenolic compounds, alkaloids, and terpenoids, which help to explain their various pharmacological effects [31].

The current study evaluates the phytochemical composition and biological activities of the methanolic extract of *A. santolina* aerial parts, with a focus on xanthine oxidase inhibition and intracellular antioxidant capacity.

LC-MS analysis revealed that the extract is mainly composed of flavonoids and phenolic acids. Most of the compounds were flavonoid glycosides, with rutin and luteolin-7-O-glucoside were the most abundant. Rutin, luteolin, and quercetin are known to have high antioxidants and enzyme inhibitory properties due to their hydroxyl-rich structures, which improve radical scavenging and metal chelation [32].

The limitation of this work is the lack of detailed MS/MS fragmentation data and confirmation using authentic reference standards, which limits the confidence level of compound identification. Future studies employing targeted MS/MS analysis and compound isolation are required to confirm the proposed structures.

The LC-MS profile revealed an unidentified chemical that accounted for 4.9% of the extract. Despite its relative abundance, this peak could not be identified due to a lack of adequate spectral matching or reference data. The presence of these compounds may contribute to reported biological activities such as xanthine oxidase inhibition and ROS modulation, either separately or in combination with other phytochemicals. Future research should focus on compound separation, purification, and complete spectroscopic characterization to identify its structure and biological function.

Although the methanolic extract of *A. santolina* contains flavonoids such as rutin, luteolin-7-O-glucoside, and quercetin, the current study did not assess these compounds individually or use activity-guided fractionation. As a result, the reported effects on xanthine oxidase inhibition and intracellular ROS modulation cannot be related to a single component. Biological activity may be due to the combined effect of several compounds, potential synergistic interactions, or the contribution of minor components. Future research involving targeted separation and quantification of the major phytochemicals, in addition to activity-guided fractionation, is needed to better understand the link between phytochemical composition and activity.

Flavonoids are recognized for their ability to regulate oxidative stress and enzyme function via various molecular mechanisms. Distinct structural characteristics, such as hydroxylation patterns, C2–C3 double bonds, and glycosylation, affect their antioxidant efficacy, enzyme binding affinity, and cellular absorption [33]. Glycosylated flavonoids typically exhibit enhanced solubility and bioavailability, although aglycones frequently demonstrate more potent direct enzyme inhibition owing to superior contact with active sites [34]. Flavonoids can inhibit intracellular reactive oxygen species (ROS) through both the scavenging of free radicals and the modulation of redox-sensitive signaling pathways and ROS-generating enzymes [35]. Formulation techniques and inherent synergy among polyphenolic elements may further augment these effects. The molecular insights affirm the biological activity of flavonoid-rich extracts, such as *A. santolina*, while highlighting that the activity shown in our work may result from various interacting pathways rather than a single compound.

These findings are consistent with previous studies on *A. santolina*. A study reported that the methanolic extract, along with its methylene chloride and butanol fractions, produced twenty-two identified compounds through HPLC analysis. Luteolin and kaempferol were isolated from the methylene chloride fraction, while isovitexin and kaempferol-3-O-glucoside were isolated from the butanol fraction [36]. Chemical profiling of petroleum ether and hydro-methanolic extracts identified epicatechin as the principal constituent in the hydro-methanolic extract, whereas camphor was the predominant compound in the petroleum ether extract [37].

On the other hand, studies on the essential oil content of *A. santolina* have also revealed significant phytochemical variation based on the extraction process and geographic origin.

Farhadi et al. [38] found that oxygenated monoterpenes such as 1,8-cineole, α-thujone, and camphor were the primary components. In Syria, the study identified camphor, eucalyptol, and terpinen-4-ol as important components [39].

Variations in phytochemical composition noted in previous research are related to alterations in extraction methods, plant provenance, and solvent polarity. These findings indicate that *Achillea* species have abundant bioactive polyphenolic compounds, likely responsible for their antioxidant and enzyme inhibitory activities.

To better position the present findings within the *Achillea* genus, comparison with existing studies is required, although the available literature remains limited. Methanolic extracts of *Achillea* species have consistently exhibited potent antioxidant and xanthine oxidase inhibitory properties, mostly due to their elevated phenolic and flavonoid concentrations. The methanolic extract of *A. filipendulina* demonstrated considerable xanthine oxidase inhibition with an IC_50_ value of 12.87 µg/mL and pronounced antioxidant activity, especially in its phenolic-rich fractions [40]. Moreover, methanolic extracts of *A. millefolium* show higher xanthine oxidase inhibitory activity (IC_50_ = 4.97 µg/mL), along with significant antioxidant and antiproliferative properties [41]. The current study presents an IC_50_ value of 29.3 µg/mL for *A. santolina*, signifying a somewhat modest potency. These variations could be linked to differences in extraction solvents, phytochemical content, and the proportion of active flavonoids.

The methanolic extract exhibited concentration-dependent inhibition of xanthine oxidase activity, albeit at a lower level than allopurinol. This discrepancy could be due to the presence of complex combinations of phytochemicals in plant extracts, as opposed to a singular pure product. The noted XO inhibition may be associated with the synergistic actions of flavonoids, including quercetin and luteolin derivatives, which have been documented to engage with the enzyme’s active site [42].

To further confirm the biological relevance of XO inhibition, intracellular ROS levels were evaluated in HepG2 cells. The administration of xanthine significantly increased ROS production, demonstrating effective induction of oxidative stress via XO-mediated mechanisms. Pre-treatment with *A. santolina* extract significantly diminished ROS formation in a dose-dependent manner. It suggests that the extract not only inhibits XO activity but also has direct antioxidant activity at the cellular level. The 24 h pre-incubation with *A. santolina* extract may facilitate both direct suppression of xanthine oxidase and indirect modification of cellular antioxidant pathways, including Nrf2-mediated defenses. The observed decrease in intracellular ROS presumably results from a combination of direct enzyme inhibition, radical scavenging, and the strengthening of endogenous antioxidant mechanisms.

The methanolic extract significantly inhibited xanthine oxidase activity and decreased intracellular ROS levels. However, the current experimental design does not prove the direct link between XO inhibition and ROS suppression in HepG2 cells. The observed reduction in ROS could be due to various mechanisms, including direct radical scavenging activity, regulation of cellular antioxidant systems, and possible XO inhibition. Additional studies, including mechanistic controls, such as allopurinol-treated cell models and cell-free ROS assessment, needed to clearly distinguish between enzyme-mediated and non-enzymatic antioxidant effects.

The reduction in reactive oxygen species may be various linked to multiple mechanisms, such as radical scavenging, inhibition of ROS-producing enzymes, and elevation of intracellular antioxidant defenses [43]. The strong relationship between phytochemical content and biological activity highlights the importance of flavonoids as main contributors to the reported effects [44].

Overall, the findings of the present study demonstrate that the methanolic extract of *A. santolina* aerial parts possesses notable xanthine oxidase inhibitory and intracellular antioxidant activities, which are strongly associated with its high content of flavonoids and phenolic compounds. The LC–MS profiling confirmed the dominance of polyphenolic constituents, while the biological assays verified their functional relevance in reducing oxidative stress through both enzyme inhibition and cellular ROS suppression.

## 4. Materials and Methods

### 4.1. Chemicals and Reagents

All chemicals and reagents utilized in this study were of analytical grade. HepG2 human hepatocellular carcinoma cells (ATCC^®^ HB-8065™) were procured from the American Type Culture Collection (ATCC, Manassas, VA, USA). The fluorescent probe 2′,7′-dichlorofluorescin diacetate (DCFH-DA), xanthine, and the xanthine oxidase inhibitor allopurinol were acquired from Sigma-Aldrich (St. Louis, MO, USA). Dulbecco’s Modified Eagle Medium (DMEM), fetal bovine serum (FBS), and penicillin-streptomycin were acquired from Gibco, Thermo Fisher Scientific (Waltham, MA, USA). Dimethyl sulfoxide (DMSO) and phosphate buffer constituents were obtained from Merck (Darmstadt, Germany). All reagents were produced in accordance with established methods.

### 4.2. Plant Material and Extraction

The aerial parts of *Achillea santolina* were collected during spring May-2025 from Amman, Jordan (32°00′54.2″ N, 35°52′22.3″ E). The species was recognized according to the descriptive reference Oran and Al-Eisawi, and a voucher specimen (No. AS-2025/11) was deposited at the Herbarium of the Department of Biological Sciences at the University of Jordan for future verification

The aerial parts of the plant were air dried until they were utterly desiccated, then ground into a fine powder. For 72 h, 20 g of powdered material were macerated in 200 mL of 80% methanol. After extraction, the mixture was filtered using Whatman filter paper (No. 1) (GE Healthcare, Chicago, IL, USA). The solvent was subsequently removed using a rotary evaporator at 50 °C. Moreover, the extracts were adequately dried and stored at 4 °C for future testing and analysis [45]. The extraction yield was calculated as 1.7% (*w*/*w*) based on the weight of the dried extract relative to the initial plant material.

### 4.3. LC-MS Analysis

The chemical constituents of the methanolic extract of *A. santolina* aerial parts were characterized using a Sciex X500R QTOF system (SCIEX, Framingham, MA, USA) in a liquid chromatography–mass spectrometry (LC–MS) analysis.

Before injection, 0.5 g of the dried methanolic extract was re-dissolved in methanol (10 mL) and filtered through a 0.22 μm syringe filter for sample preparation. Chromatographic separation was carried out on a C18 column (4.6 × 250 mm, 5 μm particle size) kept at 40 °C. The gradient program is as follows: 0–5 min, 10% B; 5–20 min, 10–90% B; 20–25 min, 90% B; and 25–30 min, re-equilibration to 10% B. The mobile phase was made up of solvents A (water comprising 0.1% formic acid) and B (acetonitrile having 0.1% formic acid). The injection volume was 10 μL, and the flow rate was 0.5 mL/min.

The following conditions have been achieved for mass spectrometric detection: ion spray voltage of 5500 V, curtain gas of 30 psi, ion source gas 1 of 50 psi, ion source gas 2 of 50 psi, and source temperature of 325 °C. The methods were positive and negative electrospray ionization (ESI). Data acquisition was performed at a scan rate of 1 scan s^−1^ on a mass range of *m*/*z* 50–1000.

Each sample was analyzed in triplicate.

Compound identification was performed based on accurate mass measurements, retention times, and comparison with spectral libraries NIST 17 Mass Spectral Library (National Institute of Standards and Technology, Gaithersburg, MD, USA), MassBank database, and SCIEX OS software (SCIEX, Framingham, MA, USA). However, MS/MS fragmentation data were not systematically acquired for all detected peaks; therefore, compound annotation should be considered tentative.

### 4.4. Xanthine Oxidase Inhibition Assay

The xanthine oxidase inhibitory activity of the methanolic extract of *A. santolina* aerial parts was assessed using the method outlined by Husnunnisa et al. [46] with slight modifications. The extract was first dissolved in dimethyl sulfoxide (DMSO) and then diluted with phosphate buffer (pH 7.5), maintaining a final DMSO content in the reaction mixture of no more than 0.1% (*v*/*v*).

Bovine milk xanthine oxidase (0.1 U/mL; Sigma-Aldrich) was used as the enzyme source. The test was performed at 25 ± 1 °C. The linearity of the reaction was confirmed by observing uric acid formation at 290 nm for a duration of 20 min (R^2^ > 0.99). Allopurinol was used as the positive control inhibitor for comparative evaluation.

The experiment was performed using 96-well microplates (SPL, Pocheon-si, Republic of Korea). Each well contained 50 µL of extract solution at different concentrations, 88 µL of phosphate buffer (pH 7.5), and 55 µL of xanthine substrate solution (0.15 mM prepared in phosphate buffer, pH 7.5). The enzymatic reaction started upon the addition of 7 µL of freshly prepared xanthine oxidase solution (0.1 U/mL). The reaction mixtures containing enzyme and substrate were incubated at 25 °C for 15 min.

The quantification of uric acid production was conducted by measuring absorbance at 290 nm with a microplate reader (BioTek Instruments ELx808, Winooski, VT, USA). All measurements were carried out in triplicate.

Control wells contained the enzyme solution without plant extract, whereas sample wells contained both the enzyme and the extract. A vehicle control containing 0.1% (*v*/*v*) DMSO without inhibitor was included to evaluate any potential effect of the solvent on xanthine oxidase activity. No significant influence of DMSO on enzyme activity was observed under the experimental conditions. To account for background absorbance, blank wells for both control and sample groups were made by substituting the enzyme solution with phosphate buffer (pH 7.5).

The percentage (%) inhibition of xanthine oxidase activity was determined using the following equation:

$$Inhibition\left(\%\right)=\frac{Absorbance\;\left(Control\right)-Absorbance\;\left(Sample\right)}{Absorbance\;(Control)}\times 100$$

A concentration–inhibition curve was made, and the IC_50_ value (the concentration required to inhibit 50% of enzyme activity) was determined by nonlinear regression analysis.

### 4.5. Reactive Oxygen Species (ROS) Assay

Human hepatocellular carcinoma cells (HepG2) were used to investigate the effect of *A. santolina* methanolic extract on intracellular reactive oxygen species (ROS) production. Cells were grown in DMEM with 10% FBS and 1% penicillin-streptomycin. They were incubated at 37 °C in a humid incubator with 5% CO_2_. Cells were cultured in ninety-six -well plates at a density of 1 × 10^4^ cells per well and allowed to adhere overnight. The cells were pre-treated with various concentrations of the methanolic extract (5, 10, 25, and 50 µg/mL) for 24 h, whereas control wells received 0.1% DMSO as a vehicle. Following pre-treatment, 0.1 mM xanthine was added to the selected wells to increase xanthine oxidase activity and ROS generation.

Intracellular reactive oxygen species levels were quantified utilizing the fluorescent probe 2′,7′-dichlorofluorescin diacetate (DCFH-DA). After the treatment period, cells were rinsed with phosphate-buffered saline and treated with 10 µM DCFH-DA in serum-free media for 30 min at 37 °C in the absence of light. The fluorescence intensity, indicative of ROS accumulation, was measured with a microplate reader at 485 nm for excitation and 535 nm for emission. Reactive oxygen species (ROS) levels were quantified as a percentage compared to untreated controls [47], and all tests were conducted in triplicate.

### 4.6. Statistical Analysis

Results are expressed as mean ± SD, with the sample size (*n* = 3). Statistical significance was determined using two-way ANOVA followed by Tukey’s post hoc test analysis conducted using GraphPad Prism 11.0.0 (GraphPad Software, San Diego, CA, USA), where *p*  <  0.05 was considered significant. Figures include error bars and significance indicators.

## 5. Conclusions

The current study shows that the methanolic extract of *A. santolina* is high in phenolic compounds. The extract inhibited xanthine oxidase and lowered intracellular ROS levels in HepG2 cells in a concentration-dependent manner. These findings underscore methanolic extract of *A. santolina*’s potential as a source of bioactive compounds with antioxidant and enzyme inhibitor activities. However, the present study is a preliminary evaluation based on crude extract analysis, and the observed biological effects cannot be linked to any specific components. Further research is required to clarify the underlying mechanisms, including bioactivity-guided fractionation, extraction and structural characterization of active compounds, comprehensive enzyme kinetics, and validation utilizing cellular pathway-specific markers. Additionally, *in vivo* or *ex vivo* experiments are required to prove the pharmacological impact and therapeutic potential of this plant.

## Figures and Tables

**Table 1:** LC–MS profile of *A. santolina* methanolic extract.

No.	Compound	Retention Time (min)	Area (%)
1	Chlorogenic acid	6.2	12.8
2	Caffeic acid	7.5	6.4
3	Unknown	9.3	4.9
4	Rutin	11.8	18.6
5	Luteolin-7-O-glucoside	13.4	15.2
6	Apigenin-7-O-glucoside	14.7	10.3
7	Quercetin	17.9	8.7
8	Luteolin	18.8	9.5
9	Apigenin	20.3	7.1
10	Kaempferol	21.6	6.5
Total identification		95.1	
Chemical classification		%	
Phenolic acid		19.2	
Flavonoid glycoside		44.1	
Flavonoid aglycone		31.8	

**Table 2:** Effect of *A. santolina* methanolic extract (ASME) on intracellular ROS production in HepG2 cells.

Treatment	ROS (%) ± SD
Control (no xanthine)	100 ± 2.1
Xanthine alone	210 ± 3.2 ***
ASME 5 µg/mL + Xanthine	172 ± 1.5 **
ASME 10 µg/mL + Xanthine	137 ± 2.0 **
ASME 25 µg/mL + Xanthine	80 ± 2.8 **
ASME 50 µg/mL + Xanthine	52 ± 3.1 **

*** *p* < 0.001 vs. control ** *p* < 0.05 vs. xanthine-treated group. Data are expressed as mean ± SD (*n* = 3).

## Data Availability

All data generated or analyzed during this study are included in this published article. Additional datasets are available from the corresponding author on reasonable request.
